# Six Essential Oils as Potential Antibacterial Agents Against Extensively Drug-Resistant (XDR) Gram-Negative Clinical Isolates

**DOI:** 10.3390/antibiotics15080782

**Published:** 2026-08-13

**Authors:** Bianca Bǎdescu-Chircea, Sergio Liga, Monica Licker, Dorina Dugăeşescu, Mihaela Diana Popa, Ciprian Nicolae Piluţ, Silvana Vulpie, Valentina Oana Buda, Delia Muntean

**Affiliations:** 1 Doctoral School, “Victor Babeș” University of Medicine and Pharmacy Timișoara, Eftimie Murgu Square No. 2, 300041 Timișoara, Romania; bianca.badescu@umft.ro; 2 Department of Microbiology, Faculty of Medicine, “Victor Babeș” University of Medicine and Pharmacy Timișoara, Eftimie Murgu Square No. 2, 300041 Timișoara, Romania; licker.monica@umft.ro (M.L.); dugaesescu.dorina@umft.ro (D.D.); popa.mihaela@umft.ro (M.D.P.); pilut.nicolae@umft.ro (C.N.P.); muntean.delia@umft.ro (D.M.); 3 Faculty of Chemical Engineering, Biotechnologies and Environmental Protection, Politehnica University Timișoara, Vasile Pârvan No. 6, 300223 Timișoara, Romania; 4 University Clinic of Toxicology, Drug Industry, Management and Legislation, Faculty of Pharmacy, “Victor Babeș” University of Medicine and Pharmacy Timișoara, Eftimie Murgu Square No. 2, 300041 Timișoara, Romania; 5 Multidisciplinary Research Center on Antimicrobial Resistance, “Victor Babeș” University of Medicine and Pharmacy Timișoara, Eftimie Murgu Square No. 2, 300041 Timișoara, Romania; 6 Microbiology Laboratory, “Pius Brînzeu” Emergency County Clinical Hospital, 300723 Timisoara, Romania; 7 Faculty of Pharmacy, “Victor Babeș” University of Medicine and Pharmacy Timișoara, Eftimie Murgu Square No. 2, 300041 Timișoara, Romania; buda.valentina@umft.ro; 8 Institute of Cardiovascular Diseases Timişoara, 300310 Timişoara, Romania; 9 Research Center for Pharmaco-Toxicological Evaluation, “Victor Babeș” University of Medicine and Pharmacy Timișoara, Eftimie Murgu Square No. 2, 300041 Timișoara, Romania

**Keywords:** essential oils, extensively drug-resistant, Gram-negative bacteria, *Satureja montana*, antibacterial activity

## Abstract

**Background/Objectives**: Extensively drug-resistant Gram-negative pathogens severely limit antimicrobial treatment options. This study evaluated the in vitro antibacterial activity of six essential oils obtained from Romanian medicinal plants against extensively drug-resistant clinical isolates and interpreted the antibacterial findings in the context of their previously reported chemical composition. **Methods**: Forty-one non-duplicate clinical isolates were included: (i) *Acinetobacter baumannii* (n = 21); (ii) *Klebsiella pneumoniae* (n = 14); (iii) *Pseudomonas aeruginosa* (n = 4); and (iv) *Providencia stuartii* (n = 2). The chemical composition of the six essential oils from *Thymus vulgaris*, *Thymus pannonicus*, *Satureja montana*, *Mentha × smithiana*, *Mentha × piperita*, and *Lavandula angustifolia* had been previously characterized by gas chromatography–mass spectrometry (GC-MS), and the corresponding compositional data were used in the present study. Antibacterial activity was assessed using disk-diffusion, disk-volatilization, and broth-dilution assays to determine minimum inhibitory and minimum bactericidal concentrations. **Results**: *Satureja montana* contained 60.9% carvacrol, *T. vulgaris* 44.6% carvacrol, and *T. pannonicus* 34.9% thymol. *S. montana* showed the strongest overall antibacterial activity, followed by *T. vulgaris* and *T. pannonicus*. *A. baumannii* isolates showed the greatest overall inhibitory response; *S. montana* produced direct-contact inhibition zones ≥ 25 mm in all 21 isolates and vapor–phase zones of 25–29 mm in 17 of 21 isolates. *K. pneumoniae* showed oil-dependent inhibition patterns, while the findings for the two *P. stuartii* isolates were interpreted descriptively because of the limited subgroup size. The four *P. aeruginosa* isolates showed the lowest overall inhibitory response, particularly in the vapor phase assay. MIC and MBC findings broadly supported the diffusion-based results, whereas *L. angustifolia* showed the weakest overall activity. **Conclusions**: Essential oils rich in phenolic monoterpenes, particularly those containing carvacrol or thymol, showed promising in vitro activity against extensively drug-resistant Gram-negative clinical isolates. These findings do not establish clinical efficacy and require confirmation in larger isolate collections using standardized assays, including cytotoxicity, biofilm, formulation, and synergy studies.

## 1. Introduction

Antimicrobial resistance among Gram-negative bacteria represents a major challenge for the management of hospital-acquired infections and increasingly limits the efficacy of conventional antibiotic therapy [1,2]. Among these microorganisms, *Acinetobacter baumannii*, *Pseudomonas aeruginosa* and *Enterobacterales*, including *Klebsiella pneumoniae* and *Providencia stuartii*, are clinically relevant because they are frequently involved in severe nosocomial infections and may accumulate multiple resistance mechanisms [1,3]. In 2017, the World Health Organization classified carbapenem-resistant *A. baumannii*, carbapenem-resistant *P. aeruginosa* and carbapenem-resistant or extended-spectrum β-lactamase-producing *Enterobacteriaceae* as critical-priority pathogens for which new antimicrobial strategies are urgently required [4,5,6]. The clinical concern is even greater when these bacteria exhibit an extensively drug-resistant (XDR) phenotype [3,7,8].

Extensively drug-resistant Gram-negative bacteria (XDR) have been increasingly reported worldwide, with *P. aeruginosa*, *A. baumannii* and *K. pneumoniae* representing the most frequently described species in published clinical reports [3,8]. These infections are usually associated with intensive care units, invasive procedures, prolonged hospitalization and previous broad-spectrum antibiotic exposure, while available therapeutic options remain extremely limited and often rely on salvage or synergistic regimens [2,3]. Therefore, the investigation of non-conventional antimicrobial agents is justified as a complementary research direction, especially for difficult-to-treat infections caused by highly resistant Gram-negative pathogens [9,10].

Essential oils, also referred to as volatile oils, are complex mixtures of low-molecular-weight bioactive compounds naturally synthesized by medicinal and aromatic plants during secondary metabolism [11]. Several essential oil components, particularly thymol and carvacrol, have been associated with antibacterial activity against Gram-negative pathogens, including multidrug-resistant clinical isolates [11,12,13]. The antibacterial activity of essential oils is mainly attributed to disruption of bacterial membrane integrity, alteration of membrane permeability, impairment of cellular respiration and leakage of intracellular components [11].

Although Gram-negative bacteria are generally less susceptible to essential oils than Gram-positive bacteria because of their outer membrane barrier, recent studies have demonstrated relevant in vitro activity of selected essential oils against multidrug-resistant Gram-negative clinical isolates [9,13,14,15]. The volatile nature of these plant-derived products also supports their evaluation not only by direct-contact methods, but also by vapor-phase assays, which may provide additional information regarding their antimicrobial potential [16]. However, data regarding the activity of essential oils against extensively drug-resistant Gram-negative clinical isolates remain limited, particularly for species such as *A. baumannii*, *K. pneumoniae*, *P. stuartii* and *P. aeruginosa* [3,9].

Therefore, the aim of the present study was to evaluate the in vitro antibacterial activity of six essential oils against a collection of XDR Gram-negative clinical isolates, including *Acinetobacter baumannii*, *Klebsiella pneumoniae*, *Providencia stuartii*, and *Pseudomonas aeruginosa*. The study extends the previously reported phytochemical characterization of these essential oils by evaluating their antibacterial activity against XDR clinical isolates.

## 2. Results

### 2.1. Previously Reported Chemical Composition of the Essential Oils

The chemical composition of the six essential oils used in the present study had been previously characterized by GC-MS and reported in earlier studies [17,18,19,20]. The corresponding compositional data are summarized in Table 1. The tested oils showed distinct phytochemical profiles, with relevant differences in the relative proportions of monoterpene hydrocarbons, oxygenated monoterpenes, phenolic monoterpenes, and sesquiterpenes.

Among the six essential oils, *Satureja montana* was characterized by the highest carvacrol content (60.9%), with γ-terpinene (9.4%) and p-cymene (8.3%) as other relevant constituents. *Thymus vulgaris* also showed a carvacrol-rich profile, with carvacrol accounting for 44.6%, followed by p-cymene (19.2%), thymol (7.6%), and γ-terpinene (4.2%). In contrast, *Thymus pannonicus* was characterized mainly by thymol (34.9%), γ-terpinene (13.9%), and p-cymene (12.0%), whereas carvacrol represented 2.3%.

The two Mentha essential oils showed markedly different compositional profiles. *Mentha* × *smithiana* was predominantly characterized by carvone (55.71%) and limonene (18.83%), followed by trans-carveol (3.54%), cis-carveol (2.72%), caryophyllene oxide (1.59%), and other minor constituents. *Mentha* × *piperita* was dominated by p-Mentha-6,8-diene-2-one (57.92%) and p-Mentha-1,8-diene (29.58%), while the remaining constituents occurred at substantially lower proportions. In the analyzed oil, menthone represented only 0.46%, while menthol was not identified among the reported constituents; instead, p-Mentha-6,8-diene-2-one (57.92%) and p-Mentha-1,8-diene (29.58%) were predominant [20]. Such differences further illustrate the compositional variability reported for Mentha essential oils [21].

*Lavandula angustifolia* showed a distinct profile, with caryophyllene as the most abundant constituent (24.12%), followed by β-phellandrene (16.00%), 1,8-cineole (15.69%), terpinen-4-ol (9.57%), α-terpineol (6.00%), and borneol (5.07%). Similarly, linalool was detected only in trace amounts (<0.05%), whereas linalyl acetate was not identified. Although these compounds are frequently reported among the characteristic constituents of *L. angustifolia* essential oils, substantial compositional variability may occur depending on genetic background, geographical origin, environmental conditions, harvesting stage, and extraction conditions [17,22].

However, because essential oil composition may vary according to plant material, harvest conditions, and analytical profile, the chemical compositional data summarized in the present study were interpreted based on the previously reported GC-MS profiles. Overall, the previously reported GC-MS profiles demonstrated substantial compositional differences among the six essential oils, which were considered when interpreting their antibacterial activity in the present study.

### 2.2. Antibacterial Activity Assessed by Disk-Diffusion and Disk-Volatilization Methods

The antibacterial activity of the six essential oils against extensively drug-resistant Gram-negative clinical isolates is presented in Table 2. Overall, the tested essential oils showed variable inhibitory activity depending on both the bacterial species and the method used for testing.

In the disk-diffusion assay, *Satureja montana* showed the most pronounced overall activity, followed by *Thymus vulgaris* and *Thymus pannonicus.* The largest inhibition zones were generally observed among the *A. baumannii* isolates, whereas the four *P. aeruginosa* isolates showed lower direct-contact inhibition. *Lavandula angustifolia* generally showed the weakest activity.

In the disk-volatilization assay, *Satureja montana*, *Thymus vulgaris*, and *Thymus pannonicus* again showed the most pronounced activity, particularly within the *A. baumannii* isolate group. In contrast, all four *P. aeruginosa* isolates showed inhibition zones ≤ 12 mm with all tested essential oils. The findings for the two *P. stuartii* isolates are reported descriptively because of the limited subgroup size. Overall, the diffusion-based assays indicated the most pronounced antibacterial activity for *Satureja montana* within the tested isolate collection.

Among the reference strains, *E. coli* ATCC 25922 generally showed larger inhibition zones, particularly with the two *Mentha* oils, whereas *P. aeruginosa* ATCC 27853 showed limited vapor-phase inhibition. The two *K. pneumoniae* reference strains showed oil-dependent responses broadly overlapping the ranges observed among the clinical *K. pneumoniae* isolates.

### 2.3. Minimum Inhibitory and Bactericidal Concentrations

Overall, the broth-dilution assay showed an activity pattern broadly consistent with the results obtained by diffusion-based methods. Among the bacterial species, *Acinetobacter baumannii* showed the most favorable MIC distribution, particularly for *Satureja montana*, *Thymus vulgaris*, and *Thymus pannonicus*. In contrast, the four *P. aeruginosa* isolates showed higher MIC and MBC values. For *K. pneumoniae* and *P. stuartii*, MIC and MBC distributions varied according to the essential oil tested. The MBC values were generally equal to or higher than the corresponding MIC values, indicating that higher concentrations were required to achieve bactericidal activity than to inhibit visible bacterial growth.

Overall, the MIC/MBC profile confirmed the strongest antibacterial activity for *Satureja montana*, followed by *Thymus vulgaris* and *Thymus pannonicus*, while *Lavandula angustifolia* showed the weakest overall activity. The MIC and MBC distributions of the tested essential oils are presented in Table 3.

## 3. Discussion

Extensively drug-resistant Gram-negative bacteria represent a clinically relevant resistance phenotype because they are associated with limited therapeutic options, frequent ICU involvement, and high reported mortality in published clinical series [3,8]. In this context, the evaluation of non-conventional antimicrobial agents is justified as an in vitro research direction, particularly when conventional antibacterial options are severely restricted [2,3]. The present study investigated six essential oils obtained from Romanian medicinal plants against clinical XDR Gram-negative isolates, focusing on *Acinetobacter baumannii*, *Klebsiella pneumoniae*, *Providencia stuartii*, and *Pseudomonas aeruginosa*.

The highest overall antibacterial activity was observed for *Satureja montana*, followed by *Thymus vulgaris* and *Thymus pannonicus*. This activity pattern appears to be consistent with the chemical composition of these oils, as *Satureja montana* and *Thymus vulgaris* were characterized by high carvacrol content, whereas *Thymus pannonicus* contained a high proportion of thymol. Phenolic monoterpenes (e.g., carvacrol, thymol) are frequently associated with strong antibacterial effects because they can interact with bacterial membranes, increase permeability, disturb membrane potential, and promote leakage of intracellular constituents [9,11]. The presence of p-cymene and γ-terpinene in these oils may also be relevant, as these compounds are commonly found in phenolic monoterpene-rich essential oils and may contribute to the overall antimicrobial profile [11]. The relevance of carvacrol and thymol in the antibacterial activity of these oils is further supported by experimental data showing that oregano essential oil and its main phenolic constituents may act by damaging membrane integrity, disrupting intracellular pH homeostasis, and disturbing the equilibrium of inorganic ions. Lambert et al. also suggested that the inhibitory effect of oregano essential oil could be largely explained by the combined activity of carvacrol and thymol, which supports the interpretation of the activity observed in the present study for *Satureja montana*, *Thymus vulgaris*, and *Thymus pannonicus* essential oils [23]. The strong activity of *Satureja montana* essential oil observed in our study is consistent with previous findings reporting carvacrol, p-cymene, and thymol as major constituents of this oil and demonstrating antimicrobial activity against both reference and clinical bacterial strains. Vitanza et al. also reported that *Satureja montana* essential oil reduced biofilm formation and induced visible bacterial morphological alterations, including cell-surface damage, which supports a membrane- and cell-envelope-targeting mechanism [24].

In contrast, *Mentha* × *piperita*, *Mentha* × *smithiana*, and *Lavandula angustifolia* showed comparatively lower antibacterial activity and markedly different phytochemical profiles from the carvacrol- and thymol-rich oils. *Mentha* × *smithiana* was dominated by carvone (55.71%) and limonene (18.83%), whereas *Mentha* × *piperita* was characterized mainly by p-Mentha-6,8-diene-2-one (57.92%) and p-Mentha-1,8-diene (29.58%). *Lavandula angustifolia* showed a distinct profile dominated by caryophyllene (24.12%), β-phellandrene (16.00%), and 1,8-cineole (15.69%). Unlike *Satureja montana* and the two *Thymus* oils, these essential oils did not contain substantial amounts of the phenolic monoterpenes carvacrol and thymol. This compositional difference may partly contribute to their lower antibacterial activity in the present isolate collection; however, essential-oil activity reflects the combined effects of multiple constituents and cannot be attributed to individual compounds based on the present study alone.

Moreover, although major constituents are often considered important contributors to the biological activity of essential oils, minor components may also influence the overall antibacterial effect, either through their individual activity or through additive, synergistic, or antagonistic interactions within the complex essential-oil mixture. Such interactions were not specifically investigated in the present study and therefore remain to be clarified in future mechanistic studies.

The contrast between phenolic-monoterpene-rich essential oils and oils lacking substantial amounts of these compounds is in line with recent data on *Lamiaceae* oils. Cagnoli et al. reported low MIC values for *Origanum vulgare*, *Satureja montana*, and *Thymus vulgaris* essential oils against pathogenic *Escherichia coli* isolates, with carvacrol and thymol representing major constituents of these oils [25]. Although that study evaluated pathogenic *E. coli* rather than XDR nosocomial isolates, it supports the broader observation that phenolic-rich essential oils from *Satureja* and *Thymus* species may show relevant activity against Gram-negative bacteria.

The differences observed between bacterial species are also biologically plausible. Among the tested isolate groups, *A. baumannii* showed the greatest overall inhibitory response, especially by *Satureja montana*, *Thymus vulgaris*, and *Thymus pannonicus*. Similar interest in essential oils against carbapenem-resistant *A. baumannii* has been reported previously, including vapor phase activity testing of *Melaleuca alternifolia* essential oil [9]. By contrast, the four *P. aeruginosa* isolates showed the lowest overall inhibitory response. The activity observed against *K. pneumoniae* varied according to the essential oil tested. The better activity of *Satureja montana*, *Thymus vulgaris*, and *Thymus pannonicus* against this species supports the relevance of phenolic-rich oils against *Enterobacterales*. However, the lower activity of *Lavandula angustifolia* and the comparatively lower activity of the two Mentha oils indicate an oil-dependent variation in antibacterial activity within the tested *K. pneumoniae* isolate collection. Because capsule expression and other strain-specific factors were not evaluated in the present study, no mechanistic inference regarding their contribution to essential-oil activity can be made.

The disk-volatilization results provided additional information regarding the antibacterial potential of the tested oils in the vapor phase. The most relevant vapor phase activity was observed for *Satureja montana*, *Thymus vulgaris*, and *Thymus pannonicus*, particularly against *A. baumannii* and *K. pneumoniae*. This finding is consistent with previous studies showing that essential oils may retain antimicrobial activity in the vapor phase and that vapor phase activity testing can complement direct-contact assays [9,16]. However, the limited vapor phase activity observed against the four *P. aeruginosa* isolates indicates that, under the experimental conditions used in the present study, vapor phase exposure produced only minimal inhibition in this isolate subgroup.

The MIC and MBC results further supported the general activity pattern observed in diffusion-based assays. The lowest inhibitory concentrations were recorded mainly for *Satureja montana, Thymus vulgaris*, and *Thymus pannonicus*, whereas *Lavandula angustifolia* showed the weakest overall inhibitory and bactericidal profile. MBC values were generally equal to or higher than MIC values, which is expected because bactericidal activity usually requires concentrations at least comparable to those needed to inhibit visible growth. These findings should be interpreted as in vitro evidence only and should not be extrapolated directly to clinical efficacy. The interpretation of MIC and MBC values for essential oils requires caution because reported values may vary according to the tested microorganism, inoculum size, culture medium, solubility conditions, emulsification procedures, and the lack of universally accepted standardized protocols and clinical breakpoints [26]. Therefore, in the present study, MIC and MBC values should be regarded as comparative in vitro indicators of antibacterial activity rather than clinically applicable susceptibility thresholds.

Overall, the present findings suggest that essential oils rich in phenolic monoterpenes, especially carvacrol and thymol, have the most promising in vitro antibacterial activity against XDR Gram-negative clinical isolates. The strongest activity of *Satureja montana* is consistent with its high carvacrol content, while the activity of *Thymus vulgaris* and *Thymus pannonicus* may be related to their carvacrol- and thymol-rich profiles. These results support further investigation of selected essential oils as complementary antimicrobial candidates, particularly in experimental models involving highly resistant Gram-negative bacteria.

## 4. Study Limitations

This study has several limitations. First, the number of isolates was limited, particularly for *Pseudomonas aeruginosa* and *Providencia stuartii*, which restricts broader species-level interpretation. Second, the present analysis was primarily descriptive, and no inferential statistical comparisons between essential oils or bacterial isolate groups were performed. Therefore, the observed differences should be interpreted as patterns within the tested isolate collection rather than as statistically confirmed differences. Third, detailed isolate-level characterization of specific resistance determinants was outside the scope of the present study; consequently, possible associations between individual resistance mechanisms and essential-oil activity were not assessed. Fourth, the study was performed in vitro, and therefore the results cannot be directly translated into clinical recommendations. Fifth, essential oils are chemically complex mixtures, and the contribution of individual compounds or synergistic interactions between constituents was not separately assessed. Sixth, although disk-volatilization testing provides useful information regarding vapor phase activity, the method is not fully standardized for clinical microbiology interpretation. Further studies using larger collections of clinical XDR isolates, standardized EO susceptibility testing protocols, time-kill assays, biofilm models, cytotoxicity testing, and pharmacological evaluation are needed before any therapeutic relevance can be proposed.

Further research should also address formulation, cytotoxicity, pharmacological feasibility, and environmental safety before any applied use of essential oils or related plant-derived volatile products can be proposed. This aspect is important because plant-derived distillation products may have antimicrobial activity but may also affect non-target microbial communities.

## 5. Materials and Methods

### 5.1. Bacterial Strains

This study included 41 non-duplicate extensively drug-resistant Gram-negative clinical isolates recovered during the routine activity of the Bacteriology Laboratory of the “Pius Brînzeu” Emergency Clinical County Hospital, Timișoara, Romania. The isolates were classified as extensively drug-resistant (XDR) according to the international consensus definitions proposed by Magiorakos et al. [7], based on acquired non-susceptibility to at least one agent in all but two or fewer antimicrobial categories.

The bacterial collection consisted of *Acinetobacter baumannii* (n = 21), *Klebsiella pneumoniae* (n = 14), *Pseudomonas aeruginosa* (n = 4), and *Providencia stuartii* (n = 2). The isolates were recovered from endotracheal aspirates (n = 34), surgical wound exudates (n = 4), blood cultures (n = 2), and urine (n = 1).

Bacterial identification was performed by matrix-assisted laser desorption ionization-time of flight mass spectrometry using the MALDI-TOF MS system (Bruker, Bremen, Germany). Antimicrobial susceptibility testing was performed by determining minimum inhibitory concentrations using the Vitek 2 Compact system (bioMérieux, Marcy-l’Étoile, France), with disk diffusion used as a complementary method [27,28]. The study was initiated in 2022. Antimicrobial susceptibility results were initially interpreted according to the CLSI standards adopted by the laboratory at that time [27]. During the study period, the laboratory transitioned to EUCAST criteria, and subsequent susceptibility results were interpreted according to the applicable EUCAST breakpoint tables [28].

The antimicrobial agents tested included ticarcillin, piperacillin, amoxicillin/clavulanic acid, ampicillin/sulbactam, ticarcillin/clavulanic acid, piperacillin/tazobactam, cefuroxime, ceftazidime, cefotaxime, ceftriaxone, ceftazidime/avibactam, ceftolozane/tazobactam, cefepime, imipenem, meropenem, imipenem/relebactam, meropenem/vaborbactam, aztreonam, amikacin, gentamicin, tobramycin, colistin, levofloxacin, moxifloxacin, ciprofloxacin, pefloxacin, tetracycline, tigecycline, minocycline, and trimethoprim/sulfamethoxazole.

Four reference strains (ThermoScientific, Waltham, MA, USA) were used for quality control and were also included in the essential-oil antibacterial testing: *Escherichia coli* ATCC 25922, *Klebsiella pneumoniae* ATCC BAA-1705, *Klebsiella pneumoniae* ATCC 700603, and *Pseudomonas aeruginosa* ATCC 27853.

### 5.2. Plant Material and Isolation of Essential Oils

The medicinal plants used for essential oil extraction originated from Romania, were harvested manually and collected at the flowering stage as follows: *Lavandula angustifolia* in July 2011; *Thymus vulgaris*, *Thymus pannonicus*, and *Satureja montana* in June–July 2012; *Mentha* × *piperita* in July–August 2017; and *Mentha* × *smithiana* in June 2019.

Botanical identification was performed before extraction, and voucher specimens were deposited in the Herbarium of the University of Life Sciences “King Mihai I” from Timisoara. Detailed botanical and phytochemical information regarding these plant materials, including GC-MS characterization of the essential oils, has been previously reported in the corresponding original publications [17,18,19,20]. Six essential oils were evaluated in the present study: *Thymus vulgaris*, *Thymus pannonicus*, *Satureja montana*, *Mentha* × *smithiana*, *Mentha* × *piperita*, and *Lavandula angustifolia* essential oils.

### 5.3. Previously Reported GC–MS Characterization of the Essential Oils

The chemical composition of the essential oils used in the present study had been previously characterized by GC–MS in the corresponding original publications [17,18,19,20]. Detailed analytical conditions, compound-identification procedures, and complete compositional profiles are provided in those studies. The previously published compositional data were summarized in Table 1 and used to provide phytochemical context for interpretation of the antibacterial activity.

### 5.4. Antibacterial Activity Assays

The antibacterial activity of the essential oils was evaluated using disk-diffusion, broth-dilution, and disk-volatilization methods. The procedures were adapted from standard antimicrobial susceptibility testing principles and from previously published studies evaluating the antibacterial activity of essential oils in liquid and vapor phases [9,16,27,28,29]. All antibacterial assays were performed in triplicate. For the disk-diffusion and disk-volatilization assays, the mean inhibition-zone diameter obtained from the three determinations was used for analysis and for assignment to the descriptive intervals presented in Table 2.

#### 5.4.1. Disk-Diffusion Method

Bacterial suspensions were prepared in 0.85% NaCl solution (bioMérieux, Marcy-l’Étoile, France) and adjusted to 0.5 McFarland using a Densimat instrument (bioMérieux, Italy). A volume of 100 μL of each bacterial suspension was inoculated onto Mueller-Hinton agar plates (bioMérieux, Marcy-l’Étoile, France). Sterile paper disks with a diameter of 6 mm (BioMaxima, Lublin, Poland) were impregnated with 10 μL of each essential oil and placed on the inoculated agar surface. The plates were incubated at 35 ± 2 °C for 18–24 h, after which the inhibition zone diameters were measured.

Inhibition-zone diameters were reported descriptively in millimeters and grouped into the intervals presented in Table 2. These intervals were used only for descriptive presentation of the results, and no clinical susceptibility categories were assigned to essential-oil activity.

#### 5.4.2. Determination of Minimum Inhibitory and Minimum Bactericidal Concentrations

The minimum inhibitory concentrations of the essential oils were determined using a broth-dilution method adapted from CLSI dilution principles [29]. The essential oils were prepared in dimethyl sulfoxide (DMSO) and tested at serial concentrations ranging from 200 to 6.25 mg/mL. Bacterial suspensions were adjusted to approximately 5 × 10^5^ CFU/mL and exposed to the corresponding essential-oil concentrations. After incubation at 35–37 °C for 24 h, the lowest essential oil concentration without visible bacterial growth was considered the MIC.

Minimum bactericidal concentrations were determined from the last two tubes showing no visible growth in the broth-dilution assay. For this purpose, 1 μL from each tube was inoculated onto Columbia agar supplemented with 5% sheep blood, and the plates were incubated overnight at 35 ± 2 °C. The MBC was defined as the lowest concentration that killed 99.9% of the initial bacterial inoculum.

#### 5.4.3. Disk-Volatilization Method

The antibacterial activity of essential oil vapors was evaluated using the disk-volatilization method described by López et al. [16]. Briefly, 100 μL of each bacterial suspension, corresponding to approximately 10^8^ CFU/mL and adjusted to 0.5 McFarland, was spread onto the surface of Mueller-Hinton agar plates.

A sterile paper disk with a diameter of 6 mm was placed on the inner surface of the Petri dish lid and impregnated with 10 μL of essential oil. The inoculated agar plate was then inverted over the lid and sealed with parafilm to prevent vapor leakage. The plates were incubated at 35 ± 2 °C for 24 h, and the inhibition zone diameters were subsequently measured.

## 6. Conclusions

The present study demonstrated that essential oils obtained from Romanian medicinal plants exhibit variable in vitro antibacterial activity against extensively drug-resistant Gram-negative clinical isolates. The antibacterial effect varied among the bacterial isolate groups. *A. baumannii* isolates showed the greatest overall inhibitory response, whereas the four *P. aeruginosa* isolates showed the lowest overall inhibitory response, particularly in the vapor phase assay. *K. pneumoniae* showed oil-dependent inhibition patterns, while the findings for the two *P. stuartii* isolates should be interpreted descriptively because of the limited subgroup size.

Among the tested oils, *Satureja montana* showed the strongest overall activity, followed by *Thymus vulgaris* and *Thymus pannonicus*, a pattern that was consistent with their high content of phenolic monoterpenes, particularly carvacrol and thymol.

The results obtained by disk-diffusion, disk-volatilization, and broth-dilution methods suggest that essential oils rich in phenolic monoterpenes may have relevant antibacterial potential against highly resistant Gram-negative bacteria. However, these findings should be interpreted strictly as in vitro evidence and cannot be directly extrapolated to clinical efficacy.

Further studies are required to confirm these results on larger collections of clinical isolates and to evaluate the mechanisms of action, antibiofilm activity, cytotoxicity, formulation stability, and potential synergistic interactions with conventional antibiotics. Standardized testing protocols for essential oils are also needed before their antimicrobial potential can be reliably compared across studies or considered for applied biomedical use.

## Figures and Tables

**Table 1:** Selected constituents of the six essential oils based on previously published GC-MS profiles (%).

Compound	*Thymus* *vulgaris*	*Thymus* *pannonicus*	*Satureja* *montana*	*Mentha* × *smithiana*	*Mentha* × *piperita*	*Lavandula* *angustifolia*
α-Pinene	0.90	0.75	0.90	0.97	1.28	0.78
Camphene	-	0.4	0.30	0.37	-	1.37
β-Pinene	0.30	0.25	0.30	0.87	1.01	0.94
3-Octanone	-	3.65	-	-	-	-
β-Myrcene	1.50	1.60	2.1	0.59	1.66	2.03
Carene	0.35	0.35	2.3	-	-	0.76
α-Phellandrene	1.05	1.35	1.65	0.45	-	-
β-Phellandrene	-	-	-	-	-	16.0
α-Terpinene	1.05	2.05	1.05	-	-	-
α-Terpineol	-	-	-	-	-	6.0
p-Cymene	19.2	12.0	8.3	0.23	-	-
Limonene	-	-	-	18.83	-	-
D-limonene	-	-	-	-	-	2.10
p-Mentha-1,8-diene	-	-	-	-	29.58	-
1,8-Cineole (Eucalyptol)	0.55	1.0	1.02	0.96	-	15.69
γ-Terpinene	4.2	13.9	9.4	-	-	0.48
Linalool	3.2	0.3	0.65	0.33	-	<0.05
Camphor	0.8	0.05	0.5	-	-	-
Borneol	1.05	0.7	0.65	0.76	-	5.07
Terpinen-4-ol	0.6	-	0.05	-	-	9.57
p-Menthan-1-ol	-	-	-	1.05	-	-
Menthone	-	-	-	-	0.46	-
Thymol methyl ether	-	3.75	-	-	-	-
Carvacrol methyl ether	-	3.45	-	-	-	-
cis-Carveol	-	-	-	2.72	-	-
trans-Carveol	-	-	-	3.54	-	-
Carvone	-	-	-	55.71	-	-
p-Mentha-6,8-diene-2-one	-	-	-	-	57.92	-
Limonene-diol	-	-	-	1.07	-	-
Thymol	7.6	34.9	-	-	-	-
Carvacrol	44.6	2.3	60.9	-	-	-
Caryophyllene	3.85	3.95	3.15	-	1.17	24.12
Caryophyllene oxide	-	4.65	-	1.59	0.21	-

**Table 2:** Antibacterial activity of the tested essential oils against extensively drug-resistant Gram-negative clinical isolates assessed by disk-diffusion and disk-volatilization methods.

Bacterial Strains	Essential Oil (EO)	Disk-Diffusion Inhibition Zones, No. of Isolates	Disk-Volatilization Inhibition Zones, No. of Isolates
*Acinetobacter baumannii* (n = 21)	*Mentha* × *piperita*	20–24 mm: 7 25–29 mm: 11 ≥30 mm: 3	15–19 mm: 4 20–24 mm: 15 25–29 mm: 2
	*Mentha* × *smithiana*	20–24 mm: 1 25–29 mm: 19 ≥30 mm: 1	15–19 mm: 2 20–24 mm: 19
	*Thymus vulgaris*	25–29 mm: 18 ≥30 mm: 3	20–24 mm: 13 25–29 mm: 8
	*Thymus pannonicus*	25–29 mm: 17 ≥30 mm: 4	15–19 mm: 1 20–24 mm: 12 25–29 mm: 8
	*Lavandula angustifolia*	20–24 mm: 19 25–29 mm: 2	15–19 mm: 12 20–24 mm: 9
	*Satureja montana*	25–29 mm: 7 ≥30 mm: 14	20–24 mm: 3 25–29 mm: 17 ≥30 mm: 1
*Klebsiella pneumoniae* (n = 14)	*Mentha* × *piperita*	15–19 mm: 10 20–24 mm: 3 25–29 mm: 1	≤12 mm: 1 13–14 mm: 4 15–19 mm: 9
	*Mentha* × *smithiana*	20–24 mm: 12 25–29 mm: 2	≤12 mm: 5 13–14 mm: 6 15–19 mm: 3
	*Thymus vulgaris*	20–24 mm: 12 25–29 mm: 2	15–19 mm: 14
	*Thymus pannonicus*	20–24 mm: 10 25–29 mm: 4	15–19 mm: 5 20–24 mm: 9
	*Lavandula angustifolia*	≤12 mm: 6 13–14 mm: 6 15–19 mm: 2	≤12 mm: 14
	*Satureja montana*	20–24 mm: 2 25–29 mm: 12	20–24 mm: 14
*Pseudomonas aeruginosa* (n = 4)	*Mentha* × *piperita*	13–14 mm: 1 15–19 mm: 2 20–24 mm: 1	≤12 mm: 4
	*Mentha* × *smithiana*	13–14 mm: 1 15–19 mm: 2 20–24 mm: 1	≤12 mm: 4
	*Thymus vulgaris*	15–19 mm: 2 20–24 mm: 2	≤12 mm: 4
	*Thymus pannonicus*	13–14 mm: 1 15–19 mm: 1 20–24 mm: 2	≤12 mm: 4
	*Lavandula angustifolia*	≤12 mm: 2 13–14 mm: 2	≤12 mm: 4
	*Satureja montana*	20–24 mm: 4	≤12 mm: 4
*Providencia stuartii* (n = 2)	*Mentha* × *piperita*	20–24 mm: 2	15–19 mm: 2
	*Mentha* × *smithiana*	20–24 mm: 2	15–19 mm: 2
	*Thymus vulgaris*	25–29 mm: 2	20–24 mm: 2
	*Thymus pannonicus*	25–29 mm: 1 ≥30 mm: 1	20–24 mm: 2
	*Lavandula angustifolia*	≤12 mm: 1 13–14 mm: 1	≤12 mm: 2
	*Satureja montana*	25–29 mm: 1 ≥30 mm: 1	20–24 mm: 1 25–29 mm: 1
*Escherichia coli* ATCC 25922	*Mentha* × *piperita*	39 mm	32 mm
	*Mentha* × *smithiana*	35 mm	31 mm
	*Thymus vulgaris*	27 mm	25 mm
	*Thymus pannonicus*	28 mm	24 mm
	*Lavandula angustifolia*	24 mm	18 mm
	*Satureja montana*	29 mm	27 mm
*Klebsiella pneumoniae* ATCC BAA-1705	*Mentha* × *piperita*	18 mm	16 mm
	*Mentha* × *smithiana*	24 mm	17 mm
	*Thymus vulgaris*	23 mm	19 mm
	*Thymus pannonicus*	22 mm	19 mm
	*Lavandula angustifolia*	13 mm	11 mm
	*Satureja montana*	28 mm	24 mm
*Klebsiella pneumoniae* ATCC 700603	*Mentha* × *piperita*	15 mm	15 mm
	*Mentha* × *smithiana*	23 mm	17 mm
	*Thymus vulgaris*	24 mm	20 mm
	*Thymus pannonicus*	20 mm	20 mm
	*Lavandula angustifolia*	12 mm	11 mm
	*Satureja montana*	30 mm	27 mm
*Pseudomonas aeruginosa* ATCC 27853	*Mentha* × *piperita*	18 mm	10 mm
	*Mentha* × *smithiana*	17 mm	10 mm
	*Thymus vulgaris*	20 mm	11 mm
	*Thymus pannonicus*	21 mm	11 mm
	*Lavandula angustifolia*	14 mm	8 mm
	*Satureja montana*	20 mm	11 mm

**Table 3:** Distribution of MIC and MBC values of the tested essential oils against extensively drug-resistant Gram-negative clinical isolates and reference strains.

Bacterial Strains	Essential Oil (EO)	MIC, No. of Isolates	MBC, No. of Isolates
*Acinetobacter baumannii* (n = 21)	*Mentha* × *piperita*	25 mg/mL: 7 12.5 mg/mL: 11 6.25 mg/mL: 3	50 mg/mL: 4 25 mg/mL: 15 12.5 mg/mL: 2
	*Mentha* × *smithiana*	25 mg/mL: 1 12.5 mg/mL: 19 6.25 mg/mL: 1	50 mg/mL: 2 25 mg/mL: 19
	*Thymus vulgaris*	12.5 mg/mL: 18 6.25 mg/mL: 3	25 mg/mL: 13 12.5 mg/mL: 8
	*Thymus pannonicus*	12.5 mg/mL: 17 6.25 mg/mL: 4	50 mg/mL: 1 25 mg/mL: 12 12.5 mg/mL: 8
	*Lavandula angustifolia*	25 mg/mL: 19 12.5 mg/mL: 2	50 mg/mL: 12 25 mg/mL: 9
	*Satureja montana*	12.5 mg/mL: 7 6.25 mg/mL: 14	25 mg/mL: 3 12.5 mg/mL: 17 6.25 mg/mL: 1
*Klebsiella pneumoniae* (n = 14)	*Mentha* × *piperita*	50 mg/mL: 10 25 mg/mL: 3 12.5 mg/mL: 1	200 mg/mL: 1 100 mg/mL: 4 50 mg/mL: 9
	*Mentha* × *smithiana*	25 mg/mL: 12 12.5 mg/mL: 2	200 mg/mL: 5 100 mg/mL: 6 50 mg/mL: 3
	*Thymus vulgaris*	25 mg/mL: 12 12.5 mg/mL: 2	50 mg/mL: 14
	*Thymus pannonicus*	25 mg/mL: 10 12.5 mg/mL: 4	50 mg/mL: 5 25 mg/mL: 9
	*Lavandula angustifolia*	200 mg/mL: 6 100 mg/mL: 6 50 mg/mL: 2	200 mg/mL: 14
	*Satureja montana*	25 mg/mL: 2 12.5 mg/mL: 12	25 mg/mL: 14
*Pseudomonas aeruginosa* (n = 4)	*Mentha* × *piperita*	100 mg/mL: 1 50 mg/mL: 2 25 mg/mL: 1	200 mg/mL: 4
	*Mentha* × *smithiana*	100 mg/mL: 1 50 mg/mL: 2 25 mg/mL: 1	200 mg/mL: 4
	*Thymus vulgaris*	50 mg/mL: 2 25 mg/mL: 2	200 mg/mL: 4
	*Thymus pannonicus*	100 mg/mL: 1 50 mg/mL: 1 25 mg/mL: 2	200 mg/mL: 4
	*Lavandula angustifolia*	200 mg/mL: 2 100 mg/mL: 2	200 mg/mL: 4
	*Satureja montana*	25 mg/mL: 4	200 mg/mL: 4
*Providencia stuartii* (n = 2)	*Mentha* × *piperita*	25 mg/mL: 2	50 mg/mL: 2
	*Mentha* × *smithiana*	25 mg/mL: 2	50 mg/mL: 2
	*Thymus vulgaris*	12.5 mg/mL: 2	25 mg/mL: 2
	*Thymus pannonicus*	12.5 mg/mL: 1 6.25 mg/mL: 1	25 mg/mL: 2
	*Lavandula angustifolia*	200 mg/mL: 1 100 mg/mL: 1	200 mg/mL: 2
	*Satureja montana*	200 mg/mL: 1 100 mg/mL: 1	200 mg/mL: 2
*Escherichia coli* ATCC 25922	*Mentha* × *piperita*	25 mg/mL	50 mg/mL
	*Mentha* × *smithiana*	25 mg/mL	50 mg/mL
	*Thymus vulgaris*	12.5 mg/mL	50 mg/mL
	*Thymus pannonicus*	12.5 mg/mL	50 mg/mL
	*Lavandula angustifolia*	100 mg/mL	200 mg/mL
	*Satureja montana*	100 mg/mL	200 mg/mL
*Klebsiella pneumoniae* ATCC BAA-1705	*Mentha* × *piperita*	50 mg/mL	100 mg/mL
	*Mentha* × *smithiana*	25 mg/mL	100 mg/mL
	*Thymus vulgaris*	25 mg/mL	50 mg/mL
	*Thymus pannonicus*	25 mg/mL	25 mg/mL
	*Lavandula angustifolia*	200 mg/mL	200 mg/mL
	*Satureja montana*	12.5 mg/mL	25 mg/mL
*Klebsiella pneumoniae* ATCC 700603	*Mentha* × *piperita*	50 mg/mL	50 mg/mL
	*Mentha* × *smithiana*	25 mg/mL	100 mg/mL
	*Thymus vulgaris*	25 mg/mL	50 mg/mL
	*Thymus pannonicus*	25 mg/mL	25 mg/mL
	*Lavandula angustifolia*	200 mg/mL	200 mg/mL
	*Satureja montana*	12.5 mg/mL	25 mg/mL
*Pseudomonas aeruginosa* ATCC 27853	*Mentha* × *piperita*	50 mg/mL	200 mg/mL
	*Mentha* × *smithiana*	50 mg/mL	200 mg/mL
	*Thymus vulgaris*	25 mg/mL	200 mg/mL
	*Thymus pannonicus*	25 mg/mL	200 mg/mL
	*Lavandula angustifolia*	100 mg/mL	200 mg/mL
	*Satureja montana*	25 mg/mL	200 mg/mL

## Data Availability

Data are contained within the article.
